# The effect of physician training and patient education on the discussion of care decisions at the internal medicine outpatient clinic

**DOI:** 10.1186/s12913-022-08901-7

**Published:** 2022-12-22

**Authors:** Saskia Briedé, Maria A. de Winter, Tessa C. van Charldorp, Karin A. H. Kaasjager

**Affiliations:** 1 Department of Internal Medicine and Dermatology, University Medical Centre Utrecht, 85500, 3508 GA Utrecht, Utrecht, The Netherlands; 2 Department of Languages, Literature and Communication, Faculty of Humanities, Utrecht University, Trans 10, 3512 JK Utrecht, Utrecht, the Netherlands

**Keywords:** Care decisions, Treatment limitations, Outpatient clinic, Provider-patient communication, Communication training, Patient education

## Abstract

**Background:**

Care decision discussions are intended to align treatment with the patient’s wishes, goals and values. To overcome the numerous barriers to such discussions, physicians as well as patients need tailored support. We evaluate the effect of a physicians’ training and a conversation aid for patients about care decisions on patient and physician outcomes.

**Methods:**

At the internal medicine outpatient clinic of the University Medical Centre Utrecht, a 1:1 randomized, parallel-group study (patient conversation aid) was combined with a pre-post intervention (physicians’ training) design. Primary outcome was patient satisfaction, secondary outcomes were patient-doctor relationship, shared-decision-making, doctor preparedness and patient appreciation of the conversation aid.

**Results:**

Between October 2018 and February 2020 11 physicians (36% residents, 73% female) and 185 patients (median age 58 years (interquartile range (IQR) 50–68), 60% male) participated. Only 28% of the patients reported a care decision discussion during the consultation. We found no effect of the interventions on patient satisfaction (effect sizes -0.14 (95% confidence interval (CI) -0.56–0.27) for conversation aid; 0.04 (95% CI -0.40–0.48) for physician’s training), nor on the patient-doctor relationship or shared-decision-making. However, physicians felt more prepared to discuss care decisions after training (median 3 (IQR 1–4) vs 1 (IQR 0–3), p = 0.015). Patients assessed the conversation aid informative and gave an overall mark of median 7 (IQR 7–8).

**Conclusions:**

First steps towards fruitful discussions about care decisions were made: patients considered the conversation aid informative and physicians felt better prepared to discuss care decisions after training. The low number of care decision conversations patients reported shows exactly how important it is to focus on interventions that facilitate these discussions, for both the patient and physician. Further work needs to be done to establish the best way to empower patients and physicians.

**Trial registration:**

Dutch trial register, trial 6998 (NTR 7188), registered 04/05/2018, https://www.trialregister.nl/trial/6998.

**Supplementary Information:**

The online version contains supplementary material available at 10.1186/s12913-022-08901-7.

## Background

The nationwide ‘Choosing Wisely’ campaign started in the USA in 2012 to engage physicians and patients in conversations about unnecessary tests, treatments and procedures, hereby contributing to appropriate healthcare [1]. Following this, the Dutch Association of Internal Medicine published a list of 10 ‘Wise Choices’. One of these ‘Wise Choices’ is to discuss care decisions when talking to patients about their treatment [2].

We define care decisions as discussions to align treatment with the patient’s wishes, goals and values, in which the option could also be to waive treatment or further investigation or to put limits to this (e.g. mechanical ventilation, dialysis, tube feeding). This includes for instance code status discussions and advanced care planning (ACP). Although the international consensus definition of ACP as posed by Rietjens et al. corresponds greatly with our vision on care decisions [3], the term ACP is strongly associated with the end of life, mostly due to the extensive research in end-of-life settings [4]. To avoid this association, we choose to use the term care decisions throughout this paper.

There are numerous barriers for both physicians and patients to discuss care decisions. Barriers for physicians described in literature are for instance: feeling unskilled or inadequately trained; discomfort and fear of complaints [5]. On top of that, physicians often wrongfully assume that patients do not want to discuss care decisions [6–8]. Patients face other difficulties, such as a lack of knowledge, unawareness of patients of the relevance, and the expectation that physicians will initiate the discussion when needed [4, 9].

When both parties avoid talking about care decisions, these discussions do not take place in time [10]. Consequently, the opportunity to adapt treatment to align with patient’s wishes is often missed [11]. Also, this results in situations in which these discussions have to be conducted in far from ideal circumstances, such as in the acute setting at the emergency department with limited time and an acutely ill patient [12]. To overcome these barriers and make way for fruitful discussions about care decisions, physicians as well as patients need tailored support.

For this study, we aimed to evaluate the effect of a training for physicians and a conversation aid for patients about the topic of care decisions. We measured patients’ and physicians’ satisfaction during the subsequent consultation at the outpatient clinic.

## Methods

### Design overview

**Fig. 1:**
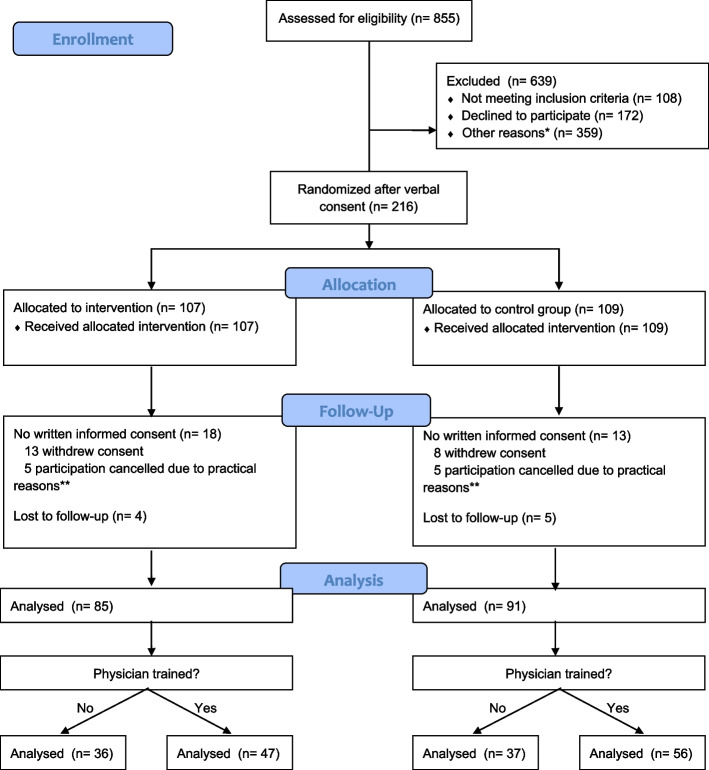
Diagram of the patient-participant flow * did not answer the phone at multiple occasions. ** consultation cancelled due to car traffic, car trouble, reschedule of the appointment for other reasons, sudden change to telephone consult

In this study, a randomized, parallel-group study was combined with a pre-post intervention design. Participating patients were randomized in a 1:1 ratio. Randomization sequence was created using Castor EDC (electronic data capture) software and was stratified by gender in a 1:1 ratio using random block sizes of 4, 6, and 8. Participating physicians were trained halfway through the study period. This resulted in 4 groups (physicians before training and patients without conversation aid (= reference group), physicians before training and patients with conversation aid, physicians after training and patients without conversation aid, physicians after training and patients with conversation aid (= intervention group) (Fig. 1). The required sample size was expected to be reached after 6–9 months, based on average number of outpatient clinic consultations per physician. 42% of eligible patient population could not be reached by phone on multiple occasions and therefore could not be approached. Furthermore, a third of the approached patients refused to participate. Due to the lower-than-expected recruitment rate and the inability to further postpone physicians’ training for logistical reasons, physicians’ training took place 7 months after the first inclusion, before half of the intended sample size was reached. The study was terminated early after 16 months because inclusion was slowing down since an increasing part of the eligible population was already approached. At this moment 80% of the attempted sample size was reached. Besides, due to the low number of actual decisions on care decisions, one of our secondary outcomes (decisional conflict) could not be properly statistically assessed. Instead, we show number of care decision discussions and decisions made. No other changes have been made to the study protocol. This study was performed in line with the principles of the Declaration of Helsinki and approved by the Medical Research Ethics Committee Utrecht (MREC 18–465) and prospectively registered 04/05/2018 at the Dutch trial register (http://www.trialregister.nl, NTR 7188).

### Setting and participants

This study was conducted at the internal medicine outpatient clinic of the University Medical Centre Utrecht (UMCU), a tertiary care teaching medical centre in the Netherlands.

#### Physicians

Eligible physicians were residents and specialists working at one of the outpatient clinics in our university hospital. Specialties at this outpatient clinic are general internal medicine, endocrinology, diabetes, nephrology, infectious diseases, immunology, vascular disease and gastroenterology. Exclusion criteria were participation in the pilot test of the e-learning module (which was used in the training), and awareness of the purpose of the study (e.g. involvement in an earlier stage or research meeting). Eligible physicians were recruited by the research team and informed that the study was about patient-doctor communication, consultations would be video-taped, and they had to fill out a questionnaire for each participating patient. They were not informed that the focus of this study was the discussion of care decisions. Written informed consent was obtained, after which their schedules were screened for eligible patients.

#### Patients

Patients ≥ 18 years with a scheduled consultation with one of the participating physicians within the study period were eligible for inclusion. The time between scheduling and the actual appointment had to be ≥ 3 weeks to account for sufficient time for the patient to consider participation and the research team had to be available to obtain written informed consent before the appointment. Visits included routine visits and new patients at the outpatient clinic that were referred by their general practitioner. They visited the outpatient clinic for a variety of indications: renal insufficiency, diabetes mellitus, hypertension, etcetera. Exclusion criteria were insufficient command of the Dutch language (i.e. unable to read and understand the conversation aid and questionnaires), inability to give informed consent and a registered discussion on treatment limitations with their physician within 2 years before the visit. Patients could only participate once.

Patients were contacted by phone by the research team and informed that the study was about patient-doctor communication, half of the participants would receive an online conversation aid and the consultation would be video-taped. They were unaware of the topic of the conversation aid and focus of the study. After verbal informed consent was obtained, participants were randomized. Patients in the control group received an e-mail with the same information as discussed during the phone call, whilst the intervention group received an e-mail with information about the topic of the conversation aid, along with the web link to the conversation aid. Written informed consent was obtained directly before the outpatient clinic consultation by the research team.

The outpatient clinic consultations were video recorded for qualitative analyses, of which the results are reported in a separate publication [13]. The video camera was visible in the consultation room and both patient and physician were aware of (and consented to) the whole consultation being video recorded.

Immediately after the consultation, both the patient and the physician received a separate questionnaire (complete questionnaires in supplementary appendix 1).

### Interventions

#### Physicians’ training

The physicians’ training consisted of an e-learning module and a hands-on training with a simulated patient (i.e. an individual trained to act as a real patient). More detailed information can be found in supplementary appendix 2. After the training, physicians were aware that care decisions were the main focus of the present study. However, physicians were instructed to do their consultations with participating patients similar to those with non-participating patients (i.e. they should not discuss care decisions solely because ‘the camera is on').

#### Patient education: conversation aid

The conversation aid for patients was an online application in which patients could find comprehensible information about why it is important to discuss care decisions, what certain treatments entail and what possible treatment limitations are. Written information was accompanied by visual material. Hyperlinks to additional information were included. The conversation aid was created in collaboration with the UMCU Patient Panel with special attention for the use of understandable language.

Due to the nature of the intervention, patients and physicians could not be blinded to their own intervention. However, both were unaware of each other’s intervention.

### Data collection and outcomes

#### Baseline characteristics

Patient characteristics were extracted from the electronic patient records by the research team (age, gender, Charlson Comorbidity Index (CCI) [14]) and collected via the patient-questionnaires (marital status, educational level, work status, health perception, quality of life and social support). Health perception and quality of life were both measured on a 11-points Likert scale (0 to 10).Social support was measured with the Oslo-3-questionairre [15], translated into Dutch using the validated forward–backward method [16, 17]. Physicians’ characteristics were collected in the physician-questionnaires (age, gender, resident/specialist and years of training or work experience).

#### Outcome measures

The primary outcome was patient satisfaction, as a mean of 2 questions of patient satisfaction on a 11-points Likert scale (0 to 10). This scale is a frequently used outcome measure for patient satisfaction in a multitude of settings and interventions [18–21]. The two questions on patient satisfaction were:

How satisfied were you with the conversation with your physician at the outpatient clinic?

How satisfied were you with the information given before, during and after your outpatient clinic visit?

Secondary outcomes were:

The patient-doctor relationship, evaluated using the Patient Doctor Relationship Questionnaire (PDRQ-9). 9 items are scored on a 5-point Likert scale ranging from 1 (very low quality) to 5 (very high quality). The total score consists of the sum of each of the items and ranges from 9 to 45 [22, 23].

Shared-decision-making, evaluated using the Shared Decision Making Questionaire-9 for physicians (SDM-Q9-DOC). 9 items are scored on a 6-point Likert scale ranging from 0 (totally disagree) to 5 (totally agree). Items are summed and multiplied by 20/9 to provide a score with 0 indicating the lowest and 100 the highest possible level of SDM [24–27].

Doctor preparedness to discuss treatment wishes, evaluated through 8 questions ranging from very generic to care decision specific, and a mock question about medication to mask the focus of this study.

Patient appreciation of the conversation aid (intervention group only), evaluated through 10 questions on aspects of the conversation aid, an overall score, and a free text space for additional suggestions.

In summary: for each patient seen by a physician, the physician needed to complete the SDM questionnaire and physician preparedness assessment, combined in one questionnaire. All patients completed the satisfaction items, and the patient-doctor relationship questionnaire. Patients in the patient intervention group additionally completed the questions on their appreciation of the conversation aid.

### Statistical analysis

We performed an intention to treat analysis. Patient characteristics are shown stratified by intervention group. Physicians’ characteristics were described narratively.

The primary outcome of mean patient satisfaction score was shown stratified by intervention using medians and interquartile ranges. The primary outcome in the intervention group (both patient and physician trained) was first compared to the reference group (neither patient nor physician trained) with a Mann–Whitney U test. Following a gatekeeping procedure to reduce the risk of a type I error, further statistical comparisons between the patient intervention-group and physician intervention-group versus the reference group would have been performed only if the primary outcome differed between the intervention group and the reference group (fixed sequence hierarchical testing). We used the same strategy for the patient-doctor relationship and shared-decision-making outcomes. To adjust for confounders while taking into account dependence between scores of patients within physicians, primary and secondary outcomes were analysed using a multilevel mixed model. Because patients within a physician might be more similar than patients from other physicians, (e.g. more satisfied, similar diseases) a random intercept for physician was added to the model. We hypothesized that the effect of the physicians training could be different for each physician due to differences in knowledge and experience, therefore we added a random slope for physicians training. Analyses were adjusted for patients’ age, gender, CCI, quality of life, and physicians’ gender and level (resident or specialist) based on previous literature [28–30]. An interaction term between patient intervention and physician intervention was added to assess whether the effect of either intervention differed depending on the other intervention. The non-significant interaction term indicated that the effects of both interventions were independent. Therefore, the interaction term was subsequently removed from further analyses and, because the sample size calculated for the fixed hierarchical testing was not met, we additionally analysed the data as being a two-by-two factorial design.

To evaluate preparedness of the physician, results of physicians’ questionnaires before and after training were compared and tested for statistical significance using Mann–Whitney U test. Patient appreciation of the conversation aid was described narratively.

All analyses were performed using IBM SPSS Statistics 25.0.0.2 software. P values < 0.05 were considered statistically significant.

### Sample size calculation

An a priori sample size calculation for the comparison of the main intervention group (physician trained, patient informed) and reference group (physician not trained, patient uninformed) on the primary outcome was performed using the statistic program G*Power. In previous studies, patient satisfaction on an 11-point Likert scale (0 to 10) was found to be between 5 and 9, with standard deviations between 1.2 and 3.2 [18–21]. Hence, we assumed the mean patient satisfaction score to be 7.0 (reference group) and 8.0 (intervention group) with a standard deviation of 2 (i.e. a Cohen’s effect size 0.5). To achieve a power of > 80% with a (one-sided) alpha of 0.05, 51 patients per group were needed. To enable stratified analysis by gender and a loss to follow-up of 10%, the required sample size would be 232 patients.

## Results

### Study participants

Eleven physicians participated in this study, including 4 residents (educational year 3–6) and 7 specialists from different areas of specialization (nephrology, vascular medicine, immunology, endocrinology, gastroenterology). The majority were female (8/11, 73%), responsible for 71% of all consultations in this study. One physician was not able to participate due to lack of time. Between October 2018 and February 2020, a total of 185 patients participated in the study. Figure 1 shows a diagram of the patient-participant flow.

The physicians’ training took place when 77 patients were included (33% of the attempted total sample size). Table 1 shows the baseline characteristics of the patients stratified by intervention group. The overall median age was 58 years (IQR 50–68), 60% were men, and the median CCI was 3.0 (IQR 1.0–5.0). A total of nine patients were lost to follow-up. These were equally divided amongst the four groups and the characteristics we had from these patients (age, gender and CCI) were similar to the overall values.

**Table 1:** Baseline characteristics of the study population

	Physician not trained, Patient uninformed *n* = 36	missing values *n* (%)	Physician not trained, Patient informed *n* = 37	missing values *n* (%)	Physician trained, Patient uninformed *n* = 56	missing values *n* (%)	Physician trained, Patient informed *n* = 47	missing values *n* (%)
Age (years)	61 (51–71)	0 (0)	63 (53–71)	0 (0)	57 (43–68)	0 (0)	56 (48–65)	0 (0)
Gender (male)	22 (61)	0 (0)	21 (57)	0 (0)	30 (54)	0 (0)	32 (68)	0 (0)
Charlson Comorbidity Index	3.5 (2.2–5.0)	0 (0)	3.0 (2.0–4.0)	0 (0)	2.5 (1.0–5.0)	0 (0)	2.0 (1.0–4.0)	0 (0)
Educational level		4 (11)		4 (11)		6 (11)		2 (4)
Primary education	3 (9)		0 (0)		4 (8)		0 (0)	
Secondary education	7 (22)		5 (15)		5 (10)		4 (9)	
Middle education	12 (38)		7 (21)		22 (44)		15 (33)	
Higher education	10 (31)		21 (64)		19 (38)		26 (58)	
Work status (working)	11 (34)	4 (11)	10 (30)	4 (11)	20 (40)	6 (11)	24 (53)	2 (4)
Children (yes)	21 (66)	4 (11)	28 (85)	4 (11)	35 (70)	6 (11)	32 (72)	2 (4)
Marital status		4 (11)		4 (11)		6 (11)		2 (4)
Single	4 (13)		3 (9)		9 (18)		5 (11)	
Married/ living with partner	24 (75)		28 (85)		33 (66)		39 (87)	
Divorced/ widowed	4 (13)		2 (6)		8 (16)		1 (2)	
OSS-3 Confidants	3.0 (2.0–4.0)	7 (19)	3.0 (3.0–4.0)	7 (19)	3.0 (2.5–4.0)	7 (13)	3.0 (3.0–4.0)	2 (4)
OSS-3 Concern	4.0 (4.0–5.0)	8 (22)	5.0 (4.0–5.0)	7 (19)	4.0 (4.0–5.0)	7 (13)	5.0 (4.0–5.0)	2 (4)
OSS-3 Neighbour’s	3.5 (2.0–5.0)	6 (17)	4.0 (3.0–5.0)	4 (11)	4.0 (3.0–5.0)	7 (13)	3.0 (3.0–4)	2 (4)
Health perception	7 (6.0–8.0)	4 (11)	6.0 (5.0–7.0)	4 (11)	7.0 (6.0–7.0)	5 (9)	7.0 (6.0–8.0)	2 (4)
Quality of life	7.5 (6.0–8.0)	4 (11)	7.0 (5.5–8.0)	4 (11)	7.0 (6.0–8.0)	6 (11)	8.0 (6.0–9.0)	2 (4)

Baseline characteristics of the patients stratified by intervention group. Data are shown as n (%) or median (IQR). For each characteristic number of missing values per intervention group are shown

Abbreviations: OSS-3: Oslo-3-questionaire for social support

### Patient satisfaction, patient-doctor relationship and shared decision making

Table 2 shows the mean patient satisfaction, patient-doctor relationship and shared-decision-making stratified by intervention group. The number of patient-reported care decision discussions during the outpatient clinic visit and in which a decision was made are shown as well. Only 45/161 (28%) patients reported to have discussed care decisions during the outpatient clinic visit, of which 25 (56%) made a decision.

**Table 2:** Patient satisfaction, patient-doctor relationship, shared decision making and number of care decision discussions per group

	Physician not trained, Patient uninformed *n* = 36	Physician not trained, Patient informed *n* = 37	Physician trained, Patient uninformed *n* = 56	Physician trained, Patient informed *n* = 47	*p*-value*
Mean patient satisfaction^a^	8.5 (8.0–9.0)	8.0 (8.0–9.0)	9.0 (8.0–9.5)	8.0 (8.0–9.3)	0.503
Patient-doctor relationship^b^	40 (36–44)	36 (34–42)	41 (34–44)	40 (36–44)	0.963
Shared decision making^c^	67 (56–77)	58 (48–73)	64 (56–73)	67 (49–76)	0.594
Care decision discussions	8/32 (25%)	8/33 (24%)	20/51 (39%)	9/45 (20%)	**
Decision made in consultation	5/8 (63%)	4/8 (50%)	12/20 (60%)	4/9 (44%)	**

Median and interquartile range. * *P*-value for difference between group “Physician not trained, Patient uninformed” and “Physician trained, Patient informed” with Mann–Whitney U test. ** not statistically analysed due to sample size

^a^scale 0–10, missing in 11/176 patients (6%)

^b^Patient Doctor Relationship Questionaire-9, scale 9–45, missing in 12/176 patients (7%)

^c^Shared Decision Making Questionnaire -9- Doctor, scale 0–100, missing in 43/176 patients (24%)

After adjusting for patient-related (age, gender, quality of life, CCI) and physician-related (specialist/resident, gender) confounders, no statistically significant association between conversation aid and physician’s training and mean satisfaction score was found (effect sizes -0.14 (95% CI -0.56 to 0.27) for conversation aid; -0.04 (95% CI -0.48 to 0.40) for physician’s training). Similarly, for the secondary outcomes, patient-doctor relationship (effect sizes -0.45 (95% CI -2.85 to 1.95) for conversation aid; 1.28 (95% CI -1.04 to 3.60) for physician’s training) and shared-decision-making (-0.01 (95% CI -5.96 to 5.94) for conversation aid; -0.23 (95% CI -8.89 to 8.42) for physician’s training) no statistically significant association was found (supplementary appendix 3). When looking at the interventions separately, no significant difference in median satisfaction score with and without the intervention was observed (before physicians’ training 8.0 (IQR 8.0–9.0), after training 8.5 (IQR 8.0–9.5), p = 0.476; without conversation aid 8.5 (IQR 8.0–9.25), with conversation aid 8.0 (IQR 8.0–9.0), p = 0.106).

### Preparedness of the physician

Physicians felt more prepared to discuss care decisions after training (median 3 (IQR 1–4) vs 1 (IQR 0–3), p = 0.015). There were no differences in general aspects of the consultation (i.e. overall satisfaction and preparedness to answer questions of the patient) or factors related to discussing care decisions between before and after the training.

### Patients appreciation of the conversation aid

Most patients appraised the conversation aid with an overall mark of 8 (median 7, IQR 7–8).

Most patients consider the conversation aid to be clear, informative, impartial, understandable and not too time-consuming. Most patients stated not to feel insecure or sad after reading the aid. When asked whether the conversation aid helped to form an opinion on care decisions, the majority of patients did not express a clear opinion.

In summary, neither of the interventions had a statistically significant effect on patient satisfaction, patient-doctor relationship and extend of shared-decision-making experienced by physicians. Physicians felt statistically significant more prepared to discuss care decisions after training and patients evaluated the conversation aid positively.

## Discussion

Neither of the interventions had a statistically significant effect on patient satisfaction, patient-doctor relationship and extent of shared decision-making experienced by physicians. Patients considered the conversation aid to be informative and easy to understand without causing insecurity or anxiety. Furthermore, physicians felt better prepared to discuss treatment decisions after the training.

With the interventions under study, we aimed to stimulate patient empowerment, patient-centred care and meaningful discussions on care decisions, all hopefully resulting in more satisfied patients. We deliberately refrained from using number of care discussions as study outcome as discussing care decisions just to ‘check off a box’ does not improve patient-centred care and leads to frustration and pressure in physicians [31]. We aimed to plant a seed, to stimulate patients to think about their preferences and to create common ground to start the conversation; not to force them to reach an immediate decision.

Although it was not the focus of our study, the low proportion of patients reporting to have discussed care decisions (28%), of which about half made a decision during the outpatient clinic visit, is remarkable. These low numbers might be explained by the perception that it is ‘too soon’ or ‘not yet relevant’ to discuss care decisions [32–34]. Besides, previous research showed physicians often miss openers from patients that could have prompted these discussions [11]. Our qualitative analysis shows that care decisions is a precarious topic for which there is no obvious interactional slot. Therefore, effort is needed to introduce the topic and create common ground [13].

There are several possible explanations for the absence of a statistically significant effect of the interventions on the mean patient satisfaction. First, we did not reach the intended sample size. This could have resulted in insufficient power to detect a possible effect. Another explanation may be the low number of care decision conversations, possibly diluting any effect. Furthermore, patient satisfaction is influenced by many factors [35]. We tried to minimize the influence of unrelated aspects by specifically directing the two questions on patient satisfaction to the conversation with the physician and the information given rather than measuring overall satisfaction, but this does not completely exclude other influences. Moreover, patient satisfaction scores without any of the interventions were high, with a median of 8.5 (IQR 8.0–9.0). It is harder to improve satisfaction, if satisfaction is already very high [36]. Finally, it could be the case that the interventions under study are not sufficient in improving care decision discussions, and therefore did not result in a statistically significant effect.

Patients’ general attitude towards the conversation aid was positive. Patients considered the conversation aid informative. Yet, they did not assess it as helpful in forming an opinion about care decisions or discussing them. A potential explanation can be that processing the information and forming an opinion requires more time, in which case the conversation aid might still have planted a seed for further consideration. Physicians are often afraid that introducing the topic of care decisions makes patients anxious or insecure [5]. It is reassuring that most patients reported that they did not feel insecure, sad or anxious when being provided with information about care decisions in the conversation aid.

Physicians indicated they felt more prepared to discuss care decisions after the training. The fact that this difference was not seen in separate important components of care decision discussions raises questions about whether the physicians actually *were* better prepared for these conversations, especially as it is known self-assessment has a poor agreement with externally assessed performances [37, 38]. However, the *feeling* of being unskilled or inadequately trained is a known barrier to discuss care decisions [5]. Therefore, we consider the *feeling* of being better prepared an important step to remove this barrier and thereby improving care decision conversations.

The strength of our study lies in the outpatient clinic setting we studied. Most research on care decisions is conducted in end-of-life settings, although it is considered essential to start discussing this in an earlier stage [2].

We are aware that our research may have several limitations. The earlier termination of the study and low participation rate could have led to selection bias. The presence of the video camera in the consultation room could have influenced the conversations and thereby patient satisfaction. A sense of familiarity between the patient and physician could have influenced care decision discussions and patient satisfaction. This ‘familiarity’ might depend on many factors (e.g. number of visits, content of those visits) which makes it impossible to control or correct for. Furthermore, the conversation aid and questionnaires were in Dutch. Our results are therefore not extendable to patients with low literacy or language barriers. Finally, reasons for why neither the physician nor the patient introduced the topic of care decisions was not asked in the questionnaires. Further work needs to be done to establish the best way to remove the remaining barriers to care decision discussions and motivate physicians and patients to engage in these discussions.

## Conclusion

Although the conversation aid for patients and training for physicians did not improve patient satisfaction in this study, these interventions can eliminate some barriers to discuss care decisions: physicians feel more prepared to discuss care decisions and patients are more informed without feeling anxious or sad. The low number of care decision discussions shows there is still a lot of work to be done. Further work needs to be done to establish the best way to remove the barriers to care decision discussions and motivate physicians and patients to engage in these discussions.

## Supplementary Information

**Additional file 1.**

**Additional file 2.**

**Additional file 3.**

## Data Availability

The datasets used and/or analysed during the current study are available from the corresponding author on reasonable request. Metadata is available in the DataverseNL repository, https://doi.org/10.34894/YFB1S1.

## Abbreviations

ACP: Advanced care planning
UMCU: University Medical Centre Utrecht
CCI: Charlson Comorbidity Index
PDRQ-9: Patient-doctor relationship questionnaire
SDM-Q9-DOC: Shared Decision Making Questionaire-9 for physicians
OSS-3: Oslo-3-questionairre
DES: Decisional Evaluation Scale
IQR: Interquartile range
