# Supplementary material for: The effect of physician training and patient education on the discussion of care decisions at the internal medicine outpatient clinic
Source: BMC Health Serv Res. 2022 Dec 22;22:1569. doi: 10.1186/s12913-022-08901-7 (PMC9773541; doi:10.1186/s12913-022-08901-7)
Supplement: Supplementary file 1 — Additional file 1. [file 12913_2022_8901_MOESM1_ESM.pdf]

Survey 'Doctor's questionnaire'

Doctor's questionnaire - Introduction

Dear colleague, Today you saw a patient at the outpatient clinic who participated in this study. The consultation was videotaped. In addition, we ask you to fill in a questionnaire about the conversation with this patient. This questionnaire first asks some general questions, followed by statements that relate to your experiences during the consultation. We ask you to indicate to what extent each statement applies.

Doctor's questionnaire - General questions

Number	Question	Answers
1.1	Date of consulationt	[ ] [ ] [ ] *(dd-mm-yyyy)*
1.2	Age	[ ]
1.3	Sex	○ Male<br>○ Female
1.4	I am...	○ Resident<br>○ Specialist
1.4.1	***If 'I am...' is equal to ''Resident' answer this question:*** Year of education	[ ]
1.4.2	***If 'I am...' is equal to 'Specialist' answer this question:*** Years of work experience as specialist	○ 0-5 years<br>○ 5-10 years<br>○ 10-15 years<br>○ 15-20 years<br>○ >20 years

Doctor's questionnaire - Research questions

Number	Question	Answers
	The following statements relate to your experience during the consultation. Can you indicate to what extent each statement applies.	
2.1	I made it clear to the patient that a decision has to be made.	◯ Fully not applicable<br>◯ Largely not applicable<br>◯ Rather not applicable<br>◯ Rather applicable<br>◯ Largely applicable<br>◯ Fully applicable
2.2	I wanted to know exactly from the patient how he/she would like to be involved in making the decision.	◯ Fully not applicable<br>◯ Largely not applicable<br>◯ Rather not applicable<br>◯ Rather applicable<br>◯ Largely applicable<br>◯ Fully applicable
2.3	I have told the patient that there are various treatment options for his/her complaints.	◯ Fully not applicable<br>◯ Largely not applicable<br>◯ Rather not applicable<br>◯ Rather applicable<br>◯ Largely applicable<br>◯ Fully applicable
2.4	I explained the advantages and disadvantages of the treatment options to the patient in detail.	◯ Fully not applicable<br>◯ Largely not applicable<br>◯ Rather not applicable<br>◯ Rather applicable<br>◯ Largely applicable<br>◯ Fully applicable

2.5	I helped the patient understand all the information.	◯ Fully not applicable<br>◯ Largely not applicable<br>◯ Rather not applicable<br>◯ Rather applicable<br>◯ Largely applicable<br>◯ Fully applicable
2.6	I asked the patient which treatment option he/she prefers.	◯ Fully not applicable<br>◯ Largely not applicable<br>◯ Rather not applicable<br>◯ Rather applicable<br>◯ Largely applicable<br>◯ Fully applicable
2.7	The patient and I have carefully considered the various treatment options.	◯ Fully not applicable<br>◯ Largely not applicable<br>◯ Rather not applicable<br>◯ Rather applicable<br>◯ Largely applicable<br>◯ Fully applicable
2.8	The patient and I selected a treatment option together.	◯ Fully not applicable<br>◯ Largely not applicable<br>◯ Rather not applicable<br>◯ Rather applicable<br>◯ Largely applicable<br>◯ Fully applicable
2.9	The patient and I have made an appointment about the further follow-up.	◯ Fully not applicable<br>◯ Largely not applicable<br>◯ Rather not applicable<br>◯ Rather applicable<br>◯ Largely applicable<br>◯ Fully applicable
2.10	I felt prepared to answer questions of the patient about the treatment.	◯ Fully not applicable<br>◯ Largely not applicable<br>◯ Rather not applicable<br>◯ Rather applicable<br>◯ Largely applicable<br>◯ Fully applicable

2.11	I gave the patient room to indicate limitations to the medical treatment.	◯ Fully not applicable<br>◯ Largely not applicable<br>◯ Rather not applicable<br>◯ Rather applicable<br>◯ Largely applicable<br>◯ Fully applicable
2.12	The patient and I spoke about what is important for their quality of life.	◯ Fully not applicable<br>◯ Largely not applicable<br>◯ Rather not applicable<br>◯ Rather applicable<br>◯ Largely applicable<br>◯ Fully applicable
2.13	I have made the patient clear that he/she is the captain of the ship.	◯ Fully not applicable<br>◯ Largely not applicable<br>◯ Rather not applicable<br>◯ Rather applicable<br>◯ Largely applicable<br>◯ Fully applicable
2.14	I felt prepared to discuss care decisions in this consult.	◯ Fully not applicable<br>◯ Largely not applicable<br>◯ Rather not applicable<br>◯ Rather applicable<br>◯ Largely applicable<br>◯ Fully applicable
2.15	In this consult I left room for the emotions of the patient.	◯ Fully not applicable<br>◯ Largely not applicable<br>◯ Rather not applicable<br>◯ Rather applicable<br>◯ Largely applicable<br>◯ Fully applicable
2.16	I have gone through the medication list (again) with the patient.	◯ Fully not applicable<br>◯ Largely not applicable<br>◯ Rather not applicable<br>◯ Rather applicable<br>◯ Largely applicable<br>◯ Fully applicable

2.17	I am satisfied with this conversation.	○ Fully not applicable<br>○ Largely not applicable<br>○ Rather not applicable<br>○ Rather applicable<br>○ Largely applicable<br>○ Fully applicable
2.18	What was the patient's role during this consultation?	○ The patient wanted to make independent decisions<br>○ The patient wanted to decide for himself, but took your opinion in serious consideration<br>○ The patient chose to take joint responsibility for the decision<br>○ The patient left the entire treatment decision to you, but informed you of his/her opinion<br>○ The patient left the entire treatment decision to you
	Thank you for completing the questionnaire! If you have any questions about this, you can contact one of the researchers at: (e-mail address removed).	

Doctor's questionnaire - Outro

Thank you for completing the questionnaire! If you have any questions about this, you can contact one of the researchers at: (e-mail address removed).

Survey 'Patient's questionnaire (controls)'

Patient's questionnaire (controls) - Satisfaction

Number	Question	Answers
1.1	Date of consultation	[ ] [ ] [ ] *(dd-mm-yyyy)*
	The first part of the questionnaire is about the conversation you had with your doctor at the outpatient clinic. As you may know, a new privacy law came into effect in May 2018. We will of course do everything we can to guarantee your privacy within this investigation. Because we think this is very important, we ask you not to fill in any traceable, personal information (such as your full name, address or telephone number) with the 'open' questions.	
1.2	How satisfied were you with your conversation with the doctor at the outpatient clinic?	○ 1 ○ 2 ○ 3 ○ 4 ○ 5 ○ 6 ○ 7 ○ 8 ○ 9 ○ 10
1.3	How satisfied were you with the information provided before, during and after your appointment at the outpatient clinic?	○ 1 ○ 2 ○ 3 ○ 4 ○ 5 ○ 6 ○ 7 ○ 8 ○ 9 ○ 10
1.4	How satisfied were you with your doctor?	○ 1 ○ 2 ○ 3 ○ 4 ○ 5 ○ 6 ○ 7 ○ 8 ○ 9 ○ 10
1.5	Room for explanation:	[ ]
	Can you indicate to what extent the following statements apply to you and your doctor at the outpatient clinic with whom you had an appointment.	
1.6	My doctor helps me.	○ not at all appropriate<br>○ somewhat appropriate<br>○ appropriate<br>○ mostly appropriate<br>○ totally appropriate
1.7	My doctor has enough time for me.	○ not at all appropriate<br>○ somewhat appropriate<br>○ appropriate<br>○ mostly appropriate<br>○ totally appropriate

1.8	I trust my doctor.	◯ not at all appropriate<br>◯ somewhat appropriate<br>◯ appropriate<br>◯ mostly appropriate<br>◯ totally appropriate
1.9	My doctor understands me.	◯ not at all appropriate<br>◯ somewhat appropriate<br>◯ appropriate<br>◯ mostly appropriate<br>◯ totally appropriate
1.10	My doctor is dedicated to help me.	◯ not at all appropriate<br>◯ somewhat appropriate<br>◯ appropriate<br>◯ mostly appropriate<br>◯ totally appropriate
1.11	My doctor and I agree on the nature of my medical symptoms.	◯ not at all appropriate<br>◯ somewhat appropriate<br>◯ appropriate<br>◯ mostly appropriate<br>◯ totally appropriate
1.12	I can talk to my doctor.	◯ not at all appropriate<br>◯ somewhat appropriate<br>◯ appropriate<br>◯ mostly appropriate<br>◯ totally appropriate
1.13	I feel content with my doctor's treatment.	◯ not at all appropriate<br>◯ somewhat appropriate<br>◯ appropriate<br>◯ mostly appropriate<br>◯ totally appropriate
1.14	I find my doctor easily accessible.	◯ not at all appropriate<br>◯ somewhat appropriate<br>◯ appropriate<br>◯ mostly appropriate<br>◯ totally appropriate

Survey 'Patient's questionnaire (controls)'

Patient's questionnaire (controls) - Care decisions

Number	Question	Answers
	You may need to be hospitalized in the future. This may be for a planned operation, or because you have suddenly become ill. There are many different treatments. Its purpose is often to cure the disease or to reduce the symptoms. We give you the best treatment available. Some treatments can be very invasive. That is why some people no longer want all treatments, for example to the intensive care unit or to be resuscitated. If you decide you no longer want a certain treatment, we call this a 'treatment limitation' or 'care decisions'.<br>The following statements are about the decision you have made (or will make) about any treatment limitations.<br>***Please indicate to what extent you agree with the following statements.***	
2.1	I know what treatment limitations are possible.	◯ Totally agree<br>◯ Agree<br>◯ Do not agree, do not disagree<br>◯ Disagree<br>◯ Totally disagree
2.2	I think I know enough about treatment limitations.	◯ Totally agree<br>◯ Agree<br>◯ Do not agree, do not disagree<br>◯ Disagree<br>◯ Totally disagree
2.3	Room for explanation:	
2.4	During the conversation with my doctor at the outpatient clinic, care decisions were also discussed.	◯ No<br>◯ Yes
2.4.1	***If 'During the conversation with my doctor at the outpatient clinic, care decisions were also discussed. is equal to 'Yes' answer this question:***<br>I myself started talking with my doctor about care decisions.	◯ No<br>◯ Yes
2.4.2	***If 'During the conversation with my doctor at the outpatient clinic, care decisions were also discussed. is equal to 'Yes' answer this question:***<br>My doctor started this conversation at the outpatient clinic about care decisions.	◯ No<br>◯ Yes
	Please indicate to what extent you agree with the following statements.	

2.4.3	***If 'During the conversation with my doctor at the outpatient clinic, care decisions were also discussed. is equal to 'Yes' answer this question:***<br>Through this conversation I am more aware that I can talk about the limits of my treatment.	○ Totally agree<br>○ Agree<br>○ Do not agree, do not disagree<br>○ Disagree<br>○ Totally disagree
2.4.4	***If 'During the conversation with my doctor at the outpatient clinic, care decisions were also discussed. is equal to 'Yes' answer this question:***<br>Through this conversation I will start talking with my family and/or friends about what is important to me and my wishes for the treatment.	○ Totally agree<br>○ Agree<br>○ Do not agree, do not disagree<br>○ Disagree<br>○ Totally disagree
2.4.5	***If 'During the conversation with my doctor at the outpatient clinic, care decisions were also discussed. is equal to 'Yes' answer this question:***<br>I am satisfied with the information I received from my doctor about care decisions.	○ Totally agree<br>○ Agree<br>○ Do not agree, do not disagree<br>○ Disagree<br>○ Totally disagree
2.4.6	***If 'During the conversation with my doctor at the outpatient clinic, care decisions were also discussed. is equal to 'Yes' answer this question:***<br>Have you made a decision about care decisions as a result of this conversation?	○ No<br>○ Yes
	Consider the moment when you have decided on any care decision. Thinking about this decision and your choice in it, how did you feel about it?	
2.4.6.2	***If 'Have you made a decision about care decisions as a result of this conversation?' is equal to 'Yes' answer this question:***<br>I am satisfied with my decision.	○ Totally agree<br>○ Agree<br>○ Do not agree, do not disagree<br>○ Disagree<br>○ Totally disagree
2.4.6.3	***If 'Have you made a decision about care decisions as a result of this conversation?' is equal to 'Yes' answer this question:***<br>I expect to stick to my choice.	○ Totally agree<br>○ Agree<br>○ Do not agree, do not disagree<br>○ Disagree<br>○ Totally disagree
2.4.6.4	***If 'Have you made a decision about care decisions as a result of this conversation?' is equal to 'Yes' answer this question:***<br>I know the pros and cons of the treatments.	○ Totally agree<br>○ Agree<br>○ Do not agree, do not disagree<br>○ Disagree<br>○ Totally disagree

2.4.6.5	***If 'Have you made a decision about care decisions as a result of this conversation?' is equal to 'Yes' answer this question:***<br>I want clearer advice.	◯ Totally agree<br>◯ Agree<br>◯ Do not agree, do not disagree<br>◯ Disagree<br>◯ Totally disagree
2.4.6.6	***If 'Have you made a decision about care decisions as a result of this conversation?' is equal to 'Yes' answer this question:***<br>I feel pressured by others about this decision.	◯ Totally agree<br>◯ Agree<br>◯ Do not agree, do not disagree<br>◯ Disagree<br>◯ Totally disagree
2.4.6.7	***If 'Have you made a decision about care decisions as a result of this conversation?' is equal to 'Yes' answer this question:***<br>I wish I could leave my decision to someone else.	◯ Totally agree<br>◯ Agree<br>◯ Do not agree, do not disagree<br>◯ Disagree<br>◯ Totally disagree
2.4.6.8	***If 'Have you made a decision about care decisions as a result of this conversation?' is equal to 'Yes' answer this question:***<br>I'm still unsure what to choose.	◯ Totally agree<br>◯ Agree<br>◯ Do not agree, do not disagree<br>◯ Disagree<br>◯ Totally disagree
2.4.6.9	***If 'Have you made a decision about care decisions as a result of this conversation?' is equal to 'Yes' answer this question:***<br>I made a well-informed choice.	◯ Totally agree<br>◯ Agree<br>◯ Do not agree, do not disagree<br>◯ Disagree<br>◯ Totally disagree
2.4.6.10	***If 'Have you made a decision about care decisions as a result of this conversation?' is equal to 'Yes' answer this question:***<br>I find it difficult to make this decision.	◯ Totally agree<br>◯ Agree<br>◯ Do not agree, do not disagree<br>◯ Disagree<br>◯ Totally disagree
2.4.6.11	***If 'Have you made a decision about care decisions as a result of this conversation?' is equal to 'Yes' answer this question:***<br>I am satisfied with the received information.	◯ Totally agree<br>◯ Agree<br>◯ Do not agree, do not disagree<br>◯ Disagree<br>◯ Totally disagree

2.4.6.12 ***If 'Have you made a decision about care decisions as a result of this conversation?' is equal to 'Yes' answer this question:***
My choice scares me.

- ◯ Totally agree
- ◯ Agree
- ◯ Do not agree, do not disagree
- ◯ Disagree
- ◯ Totally disagree

2.4.6.13 ***If 'Have you made a decision about care decisions as a result of this conversation?' is equal to 'Yes' answer this question:***
I want more information about this choice.

- ◯ Totally agree
- ◯ Agree
- ◯ Do not agree, do not disagree
- ◯ Disagree
- ◯ Totally disagree

2.4.6.14 ***If 'Have you made a decision about care decisions as a result of this conversation?' is equal to 'Yes' answer this question:***
This decision is beyond me.

- ◯ Totally agree
- ◯ Agree
- ◯ Do not agree, do not disagree
- ◯ Disagree
- ◯ Totally disagree

2.4.6.15 ***If 'Have you made a decision about care decisions as a result of this conversation?' is equal to 'Yes' answer this question:***
I regret my choice.

- ◯ Totally agree
- ◯ Agree
- ◯ Do not agree, do not disagree
- ◯ Disagree
- ◯ Totally disagree

2.4.6.16 ***If 'Have you made a decision about care decisions as a result of this conversation?' is equal to 'Yes' answer this question:***
I am shocked by the conversation about care decisions.

- ◯ Totally agree
- ◯ Agree
- ◯ Do not agree, do not disagree
- ◯ Disagree
- ◯ Totally disagree

2.4.6.17 ***If 'Have you made a decision about care decisions as a result of this conversation?' is equal to 'Yes' answer this question:***
What questions do you still have? What are you concerned about?

Consider the moment when you have decided on any care decision. Thinking about this decision and your choice in it, how did you feel about it?

2.4.6.19 ***If 'Have you made a decision about care decisions as a result of this conversation?' is equal to 'No' answer this question:***
I am shocked by the conversation about care decisions.

- ◯ Totally agree
- ◯ Agree
- ◯ Do not agree, do not disagree
- ◯ Disagree
- ◯ Totally disagree

2.4.6.20	***If 'Have you made a decision about care decisions as a result of this conversation?' is equal to 'No' answer this question:***<br>I'm still unsure what to choose.	◯ Totally agree<br>◯ Agree<br>◯ Do not agree, do not disagree<br>◯ Disagree<br>◯ Totally disagree
2.4.6.21	***If 'Have you made a decision about care decisions as a result of this conversation?' is equal to 'No' answer this question:***<br>I find it difficult to make this decision.	◯ Totally agree<br>◯ Agree<br>◯ Do not agree, do not disagree<br>◯ Disagree<br>◯ Totally disagree
2.4.6.22	***If 'Have you made a decision about care decisions as a result of this conversation?' is equal to 'No' answer this question:***<br>I know the pros and cons of the treatments.	◯ Totally agree<br>◯ Agree<br>◯ Do not agree, do not disagree<br>◯ Disagree<br>◯ Totally disagree
2.4.6.23	***If 'Have you made a decision about care decisions as a result of this conversation?' is equal to 'No' answer this question:***<br>I am satisfied with the received information.	◯ Totally agree<br>◯ Agree<br>◯ Do not agree, do not disagree<br>◯ Disagree<br>◯ Totally disagree
2.4.6.24	***If 'Have you made a decision about care decisions as a result of this conversation?' is equal to 'No' answer this question:***<br>I want clearer advice.	◯ Totally agree<br>◯ Agree<br>◯ Do not agree, do not disagree<br>◯ Disagree<br>◯ Totally disagree
2.4.6.25	***If 'Have you made a decision about care decisions as a result of this conversation?' is equal to 'No' answer this question:***<br>I want more information about this.	◯ Totally agree<br>◯ Agree<br>◯ Do not agree, do not disagree<br>◯ Disagree<br>◯ Totally disagree
2.4.6.26	***If 'Have you made a decision about care decisions as a result of this conversation?' is equal to 'No' answer this question:***<br>I feel pressured by others about this decision.	◯ Totally agree<br>◯ Agree<br>◯ Do not agree, do not disagree<br>◯ Disagree<br>◯ Totally disagree

2.4.6.27	***If 'Have you made a decision about care decisions as a result of this conversation?' is equal to 'No' answer this question:***<br>Talking about care decisions scares me.	◯ Totally agree<br>◯ Agree<br>◯ Do not agree, do not disagree<br>◯ Disagree<br>◯ Totally disagree
2.4.6.28	***If 'Have you made a decision about care decisions as a result of this conversation?' is equal to 'No' answer this question:***<br>This decision is beyond me.	◯ Totally agree<br>◯ Agree<br>◯ Do not agree, do not disagree<br>◯ Disagree<br>◯ Totally disagree
2.4.6.29	***If 'Have you made a decision about care decisions as a result of this conversation?' is equal to 'No' answer this question:***<br>I wish I could leave my decision to someone else.	◯ Totally agree<br>◯ Agree<br>◯ Do not agree, do not disagree<br>◯ Disagree<br>◯ Totally disagree
2.4.6.30	***If 'Have you made a decision about care decisions as a result of this conversation?' is equal to 'No' answer this question:***<br>I wish I could leave my decision to someone else.	◯ Totally agree<br>◯ Agree<br>◯ Do not agree, do not disagree<br>◯ Disagree<br>◯ Totally disagree
	You indicate that care decisions were not discussed with your doctor. What about that is true for you?	
2.4.8	***If 'During the conversation with my doctor at the outpatient clinic, care decisions were also discussed. is equal to 'No' answer this question:***<br>I have already thought about care decisions myself.	◯ Totally agree<br>◯ Agree<br>◯ Do not agree, do not disagree<br>◯ Disagree<br>◯ Totally disagree
2.4.9	***If 'During the conversation with my doctor at the outpatient clinic, care decisions were also discussed. is equal to 'No' answer this question:***<br>I would have liked to discuss care decisions with my doctor.	◯ Totally agree<br>◯ Agree<br>◯ Do not agree, do not disagree<br>◯ Disagree<br>◯ Totally disagree
2.4.10	***If 'During the conversation with my doctor at the outpatient clinic, care decisions were also discussed. is equal to 'No' answer this question:***<br>I did not dare to introduce care decisions with my doctor.	◯ Totally agree<br>◯ Agree<br>◯ Do not agree, do not disagree<br>◯ Disagree<br>◯ Totally disagree

2.4.11 ***If 'During the conversation with my doctor at the outpatient clinic, care decisions were also discussed. is equal to 'No' answer this question:***
Room for explanation:

If you have any questions about care decisions as a result of this questionnaire, you can discuss this with your doctor at the outpatient clinic at the next appointment. You can also contact the researchers via (e-mail address removed), or the independent physician via (e-mail address removed) or via (telephone number removed).

Survey 'Patient's questionnaire (controls)'

Patient's questionnaire (controls) - Health perception and mood.

Number	Question	Answers
	The following questions are about how you assess your health. The answers help to keep track of how you are feeling and how well you are able to perform your usual activities.	
3.1	How do you view your health?	◯ Good<br>◯ Reasonable<br>◯ Neutral<br>◯ Moderate<br>◯ Bad
3.2	How do you experience your health?	◯ 1 ◯ 2 ◯ 3 ◯ 4 ◯ 5 ◯ 6 ◯ 7<br>◯ 8 ◯ 9 ◯ 10
3.3	How do you experience your quality of life?	◯ 1 ◯ 2 ◯ 3 ◯ 4 ◯ 5 ◯ 6 ◯ 7<br>◯ 8 ◯ 9 ◯ 10
3.4	How much are you limited in your work or other daily activities by your physical health?	◯ 1 ◯ 2 ◯ 3 ◯ 4 ◯ 5 ◯ 6 ◯ 7<br>◯ 8 ◯ 9 ◯ 10
3.5	How much are you limited in your work or other daily activities by your emotional health?	◯ 1 ◯ 2 ◯ 3 ◯ 4 ◯ 5 ◯ 6 ◯ 7<br>◯ 8 ◯ 9 ◯ 10
3.6	In the past 2 weeks, how often have you suffered from one or more of the following problems?<br>Little interest or pleasure in activities.	◯ Not at all<br>◯ Multiple days<br>◯ More than half of the days<br>◯ Almost every day
3.7	In the past 2 weeks, how often have you suffered from one or more of the following problems? Feeling down, depressed, or desperate.	◯ Not at all<br>◯ Multiple days<br>◯ More than half of the days<br>◯ Almost every day
3.8	Can you turn to your neighbors if you need help?	◯ Not at all<br>◯ Barely<br>◯ Maybe<br>◯ Reasonable<br>◯ Certainly

3.9	How many people can you contact if you have serious problems?	○ None<br>○ 1-2 people<br>○ 3-5 people<br>○ 6 or more people
3.10	How much involvement and interest do people show in what you do?	○ A lot<br>○ A little<br>○ Unclear<br>○ Few<br>○ None

Patient's questionnaire (controls) - General questions

Number	Question	Answers
4.1	What is your marital status?	◯ Single<br>◯ Living together<br>◯ Married<br>◯ Divorced<br>◯ Widow or widower
4.2	Do you have any children?	◯No<br>◯Yes
4.2.1	***If 'Do you have any children?' is equal to 'Yes' answer this question:***<br>How many children do you have?	[ ] children
4.3	What is your highest level of completed education?	◯ Primary education (Basisonderwijs)<br>◯ Secondary education (Vmbo of onderbouw havo-vwo)<br>◯ Middle education (MBO of bovenbouw havo-vwo)<br>◯ Higher education (HBO of WO bachelor)<br>◯ Higher education (Dutch: WO master)
4.4	Are you currently working?	◯ No<br>◯ Yes
4.4.1	***If 'Are you currently working?' is equal to 'Yes' answer this question:***<br>How many hours per week do you work on average?	[ ]
4.4.2	***If 'Are you currently working?' is equal to 'No' answer this question:***<br>Explanation:	◯ Retired<br>◯ Disabled<br>◯ Unemployed<br>◯ Other

This is the end of the questionnaire. This is also the last part of this research. If you have any questions about the study as a result of the questionnaire, you can contact the researchers via (e-mail address removed), or the independent physician, via (e-mailaddress removed) or via (telephone number removed).

Survey 'Patient's questionnaire (intervention)'

Patient's questionnaire (intervention) - Satisfaction

Number	Question	Answers
1.1	Date of consultation	☐ ☐ ☐ *(dd-mm-yyyy)*
	The first part of the questionnaire is about the conversation you had with your doctor at the outpatient clinic.	
1.2	How satisfied were you with your conversation with the doctor at the outpatient clinic?	○ 1 ○ 2 ○ 3 ○ 4 ○ 5 ○ 6 ○ 7 ○ 8 ○ 9 ○ 10
1.3	How satisfied were you with the information provided before, during and after your appointment at the outpatient clinic?	○ 1 ○ 2 ○ 3 ○ 4 ○ 5 ○ 6 ○ 7 ○ 8 ○ 9 ○ 10
1.4	Room for explanation:	☐
	Can you indicate to what extent the following statements apply to you and your doctor at the outpatient clinic with whom you had an appointment.	
1.5	My doctor helps me.	○ not at all appropriate<br>○ somewhat appropriate<br>○ appropriate<br>○ mostly appropriate<br>○ totally appropriate
1.6	My doctor has enough time for me.	○ not at all appropriate<br>○ somewhat appropriate<br>○ appropriate<br>○ mostly appropriate<br>○ totally appropriate
1.7	I trust my doctor.	○ not at all appropriate<br>○ somewhat appropriate<br>○ appropriate<br>○ mostly appropriate<br>○ totally appropriate

1.8	My doctor understands me.	◯ not at all appropriate<br>◯ somewhat appropriate<br>◯ appropriate<br>◯ mostly appropriate<br>◯ totally appropriate
1.9	My doctor is dedicated to help me.	◯ not at all appropriate<br>◯ somewhat appropriate<br>◯ appropriate<br>◯ mostly appropriate<br>◯ totally appropriate
1.10	My doctor and I agree on the nature of my medical symptoms.	◯ not at all appropriate<br>◯ somewhat appropriate<br>◯ appropriate<br>◯ mostly appropriate<br>◯ totally appropriate
1.11	I can talk to my doctor.	◯ not at all appropriate<br>◯ somewhat appropriate<br>◯ appropriate<br>◯ mostly appropriate<br>◯ totally appropriate
1.12	I feel content with my doctor's treatment.	◯ not at all appropriate<br>◯ somewhat appropriate<br>◯ appropriate<br>◯ mostly appropriate<br>◯ totally appropriate
1.13	I find my doctor easily accessible.	◯ not at all appropriate<br>◯ somewhat appropriate<br>◯ appropriate<br>◯ mostly appropriate<br>◯ totally appropriate

Survey 'Patient's questionnaire (intervention)'

Patient's questionnaire (intervention) - Care decisions

Number	Question	Answers
	You may need to be hospitalized in the future. This may be for a planned operation, or because you have suddenly become ill. There are many different treatments. Its purpose is often to cure the disease or to reduce the symptoms. We give you the best treatment available. Some treatments can be very invasive. That is why some people no longer want all treatments, for example to the intensive care unit or to be resuscitated. If you decide you no longer want a certain treatment, we call this a 'treatment limitation'.<br>The following statements are about the decision you have made (or will make) about any treatment limitations.<br>***Please indicate to what extent you agree with the following statements.***	
2.1	I know what treatment limitations are possible.	◯ Totally agree<br>◯ Agree<br>◯ Do not agree, do not disagree<br>◯ Disagree<br>◯ Totally disagree
2.2	I think I know enough about treatment limitations.	◯ Totally agree<br>◯ Agree<br>◯ Do not agree, do not disagree<br>◯ Disagree<br>◯ Totally disagree
2.3	Room for explanation:	
2.4	During the conversation with my doctor at the outpatient clinic, care decisions were also discussed.	◯ No<br>◯ Yes
2.4.1	***If 'During the conversation with my doctor at the outpatient clinic, care decisions were also discussed. is equal to 'Yes' answer this question:***<br>I myself started talking with my doctor about care decisions.	◯ No<br>◯ Yes
2.4.2	***If 'During the conversation with my doctor at the outpatient clinic, care decisions were also discussed. is equal to 'Yes' answer this question:***<br>My doctor started this conversation at the outpatient clinic about care decisions.	◯ No<br>◯ Yes
	Please indicate to what extent you agree with the following statements.	

2.4.3	***If 'During the conversation with my doctor at the outpatient clinic, care decisions were also discussed. is equal to 'Yes' answer this question:***<br>Through this conversation I am more aware that I can talk about the limits of my treatment.	◯ Totally agree<br>◯ Agree<br>◯ Do not agree, do not disagree<br>◯ Disagree<br>◯ Totally disagree
2.4.4	***If 'During the conversation with my doctor at the outpatient clinic, care decisions were also discussed. is equal to 'Yes' answer this question:***<br>Through this conversation I will start talking with my family and/or friends about what is important to me and my wishes for the treatment.	◯ Totally agree<br>◯ Agree<br>◯ Do not agree, do not disagree<br>◯ Disagree<br>◯ Totally disagree
2.4.5	***If 'During the conversation with my doctor at the outpatient clinic, care decisions were also discussed. is equal to 'Yes' answer this question:***<br>I am satisfied with the information I received from my doctor about care decisions.	◯ Totally agree<br>◯ Agree<br>◯ Do not agree, do not disagree<br>◯ Disagree<br>◯ Totally disagree
2.4.6	***If 'During the conversation with my doctor at the outpatient clinic, care decisions were also discussed. is equal to 'Yes' answer this question:***<br>Have you made a decision about care decisions as a result of this conversation?	◯ No<br>◯ Yes
	Consider the moment when you have decided on any care decision. Thinking about this decision and your choice in it, how did you feel about it?	
2.4.6.2	***If 'Have you made a decision about care decisions as a result of this conversation?' is equal to 'Yes' answer this question:***<br>I am satisfied with my decision.	◯ Totally agree<br>◯ Agree<br>◯ Do not agree, do not disagree<br>◯ Disagree<br>◯ Totally disagree
2.4.6.3	***If 'Have you made a decision about care decisions as a result of this conversation?' is equal to 'Yes' answer this question:***<br>I expect to stick to my choice.	◯ Totally agree<br>◯ Agree<br>◯ Do not agree, do not disagree<br>◯ Disagree<br>◯ Totally disagree
2.4.6.4	***If 'Have you made a decision about care decisions as a result of this conversation?' is equal to 'Yes' answer this question:***<br>I know the pros and cons of the treatments.	◯ Totally agree<br>◯ Agree<br>◯ Do not agree, do not disagree<br>◯ Disagree<br>◯ Totally disagree

2.4.6.5	***If 'Have you made a decision about care decisions as a result of this conversation?' is equal to 'Yes' answer this question:***<br>I want clearer advice.	◯ Totally agree<br>◯ Agree<br>◯ Do not agree, do not disagree<br>◯ Disagree<br>◯ Totally disagree
2.4.6.6	***If 'Have you made a decision about care decisions as a result of this conversation?' is equal to 'Yes' answer this question:***<br>I feel pressured by others about this decision.	◯ Totally agree<br>◯ Agree<br>◯ Do not agree, do not disagree<br>◯ Disagree<br>◯ Totally disagree
2.4.6.7	***If 'Have you made a decision about care decisions as a result of this conversation?' is equal to 'Yes' answer this question:***<br>If wish I could leave my decision to someone else.	◯ Totally agree<br>◯ Agree<br>◯ Do not agree, do not disagree<br>◯ Disagree<br>◯ Totally disagree
2.4.6.8	***If 'Have you made a decision about care decisions as a result of this conversation?' is equal to 'Yes' answer this question:***<br>I'm still unsure what to choose.	◯ Totally agree<br>◯ Agree<br>◯ Do not agree, do not disagree<br>◯ Disagree<br>◯ Totally disagree
2.4.6.9	***If 'Have you made a decision about care decisions as a result of this conversation?' is equal to 'Yes' answer this question:***<br>I made a well-informed choice.	◯ Totally agree<br>◯ Agree<br>◯ Do not agree, do not disagree<br>◯ Disagree<br>◯ Totally disagree
2.4.6.10	***If 'Have you made a decision about care decisions as a result of this conversation?' is equal to 'Yes' answer this question:***<br>I find it difficult to make this decision.	◯ Totally agree<br>◯ Agree<br>◯ Do not agree, do not disagree<br>◯ Disagree<br>◯ Totally disagree
2.4.6.11	***If 'Have you made a decision about care decisions as a result of this conversation?' is equal to 'Yes' answer this question:***<br>I am satisfied with the received information.	◯ Totally agree<br>◯ Agree<br>◯ Do not agree, do not disagree<br>◯ Disagree<br>◯ Totally disagree

2.4.6.12	***If 'Have you made a decision about care decisions as a result of this conversation?' is equal to 'Yes' answer this question:***<br>My choice scares me.	○ Totally agree<br>○ Agree<br>○ Do not agree, do not disagree<br>○ Disagree<br>○ Totally disagree
2.4.6.13	***If 'Have you made a decision about care decisions as a result of this conversation?' is equal to 'Yes' answer this question:***<br>I want more information about this choice.	○ Totally agree<br>○ Agree<br>○ Do not agree, do not disagree<br>○ Disagree<br>○ Totally disagree
2.4.6.14	***If 'Have you made a decision about care decisions as a result of this conversation?' is equal to 'Yes' answer this question:***<br>This decision is beyond me.	○ Totally agree<br>○ Agree<br>○ Do not agree, do not disagree<br>○ Disagree<br>○ Totally disagree
2.4.6.15	***If 'Have you made a decision about care decisions as a result of this conversation?' is equal to 'Yes' answer this question:***<br>I regret my choice.	○ Totally agree<br>○ Agree<br>○ Do not agree, do not disagree<br>○ Disagree<br>○ Totally disagree
2.4.6.16	***If 'Have you made a decision about care decisions as a result of this conversation?' is equal to 'Yes' answer this question:***<br>I am shocked by the conversation about care decisions.	○ Totally agree<br>○ Agree<br>○ Do not agree, do not disagree<br>○ Disagree<br>○ Totally disagree
2.4.6.17	***If 'Have you made a decision about care decisions as a result of this conversation?' is equal to 'Yes' answer this question:***<br>What questions do you still have? What are you concerned about?	
	Consider the moment when you have decided on any care decision. Thinking about this decision and your choice in it, how did you feel about it?	
2.4.6.19	***If 'Have you made a decision about care decisions as a result of this conversation?' is equal to 'No' answer this question:***<br>I am shocked by the conversation about care decisions.	○ Totally agree<br>○ Agree<br>○ Do not agree, do not disagree<br>○ Disagree<br>○ Totally disagree

2.4.6.20	***If 'Have you made a decision about care decisions as a result of this conversation?' is equal to 'No' answer this question:***<br>I'm still unsure what to choose.	◯ Totally agree<br>◯ Agree<br>◯ Do not agree, do not disagree<br>◯ Disagree<br>◯ Totally disagree
2.4.6.21	***If 'Have you made a decision about care decisions as a result of this conversation?' is equal to 'No' answer this question:***<br>I find it difficult to make this decision.	◯ Totally agree<br>◯ Agree<br>◯ Do not agree, do not disagree<br>◯ Disagree<br>◯ Totally disagree
2.4.6.22	***If 'Have you made a decision about care decisions as a result of this conversation?' is equal to 'No' answer this question:***<br>I know the pros and cons of the treatments.	◯ Totally agree<br>◯ Agree<br>◯ Do not agree, do not disagree<br>◯ Disagree<br>◯ Totally disagree
2.4.6.23	***If 'Have you made a decision about care decisions as a result of this conversation?' is equal to 'No' answer this question:***<br>I am satisfied with the received information.	◯ Totally agree<br>◯ Agree<br>◯ Do not agree, do not disagree<br>◯ Disagree<br>◯ Totally disagree
2.4.6.24	***If 'Have you made a decision about care decisions as a result of this conversation?' is equal to 'No' answer this question:***<br>I want clearer advice.	◯ Totally agree<br>◯ Agree<br>◯ Do not agree, do not disagree<br>◯ Disagree<br>◯ Totally disagree
2.4.6.25	***If 'Have you made a decision about care decisions as a result of this conversation?' is equal to 'No' answer this question:***<br>I want more information about this.	◯ Totally agree<br>◯ Agree<br>◯ Do not agree, do not disagree<br>◯ Disagree<br>◯ Totally disagree
2.4.6.26	***If 'Have you made a decision about care decisions as a result of this conversation?' is equal to 'No' answer this question:***<br>I feel pressured by others about this decision.	◯ Totally agree<br>◯ Agree<br>◯ Do not agree, do not disagree<br>◯ Disagree<br>◯ Totally disagree

2.4.6.27 ***If 'Have you made a decision about care decisions as a result of this conversation?' is equal to 'No' answer this question:***
Talking about care decisions scares me.

- ◯ Totally agree
- ◯ Agree
- ◯ Do not agree, do not disagree
- ◯ Disagree
- ◯ Totally disagree

2.4.6.28 ***If 'Have you made a decision about care decisions as a result of this conversation?' is equal to 'No' answer this question:***
This decision is beyond me.

- ◯ Totally agree
- ◯ Agree
- ◯ Do not agree, do not disagree
- ◯ Disagree
- ◯ Totally disagree

2.4.6.29 ***If 'Have you made a decision about care decisions as a result of this conversation?' is equal to 'No' answer this question:***
I wish I could leave my decision to someone else.

- ◯ Totally agree
- ◯ Agree
- ◯ Do not agree, do not disagree
- ◯ Disagree
- ◯ Totally disagree

2.4.6.30 ***If 'Have you made a decision about care decisions as a result of this conversation?' is equal to 'No' answer this question:***
I wish I could leave my decision to someone else.

- ◯ Totally agree
- ◯ Agree
- ◯ Do not agree, do not disagree
- ◯ Disagree
- ◯ Totally disagree

You indicate that care decisions were not discussed with your doctor. What about that is true for you?

2.4.8 ***If 'During the conversation with my doctor at the outpatient clinic, care decisions were also discussed. is equal to 'No' answer this question:***
I have already thought about care decisions myself.

- ◯ Totally agree
- ◯ Agree
- ◯ Do not agree, do not disagree
- ◯ Disagree
- ◯ Totally disagree

2.4.9 ***If 'During the conversation with my doctor at the outpatient clinic, care decisions were also discussed. is equal to 'No' answer this question:***
I would have liked to discuss care decisions with my doctor.

- ◯ Totally agree
- ◯ Agree
- ◯ Do not agree, do not disagree
- ◯ Disagree
- ◯ Totally disagree

2.4.10 ***If 'During the conversation with my doctor at the outpatient clinic, care decisions were also discussed. is equal to 'No' answer this question:***
I did not dare to introduce care decisions with my doctor.

- ◯ Totally agree
- ◯ Agree
- ◯ Do not agree, do not disagree
- ◯ Disagree
- ◯ Totally disagree

2.4.11 ***If 'During the conversation with my doctor at the outpatient clinic, care decisions were also discussed. is equal to 'No' answer this question:***
Room for explanation:

If you have any questions about care decisions as a result of this questionnaire, you can discuss this with your doctor at the outpatient clinic at the next appointment. You can also contact the researchers via (e-mail address removed), or the independent physician via (e-mail address removed) or via (telephone number removed).

Survey 'Patient's questionnaire (intervention)'

Patient's questionnaire (intervention) - Conversation aid

Number	Question	Answers
	Half of the people participating in this study received a conversation aid about care decisions. You have also received this preparation. The following questions are about the conversation aid you have received.	
3.1	Have you read the conversation aid?	◯ No<br>◯ Yes
3.1.1	***If 'Have you read the conversation aid?' is equal to 'No' answer this question:***<br>Why did you not read the conversation aid?	☐ I forgot to read the conversation aid.<br>☐ I could not find the conversation aid.<br>☐ I found the conversation aid to difficult.<br>☐ I did not feel comfortable reading about this topic.<br>☐ This topic scares me.<br>☐ I did not think the conversation aid would be helpful for me.<br>☐ I thought reading it would take to mucht time.<br>☐ I already know enough about care decisions.<br>☐ I had to little motivation to read the conversation aid<br>☐ Other reason
3.1.1.1	***If 'Why did you not read the conversation aid?' is equal to 'Other reason' answer this question:***<br>What is the reason that you have not read the conversation aid?	
	Please indicate to what extent you agree with the following statements.	
3.1.3	***If 'Have you read the conversation aid?' is equal to 'Yes' answer this question:***<br>The conversation aid is clear.	◯ Totally agree<br>◯ Agree<br>◯ Do not agree, do not disagree<br>◯ Disagree<br>◯ Totally disagree
3.1.4	***If 'Have you read the conversation aid?' is equal to 'Yes' answer this question:***<br>The conversation aid provides enough information.	◯ Totally agree<br>◯ Agree<br>◯ Do not agree, do not disagree<br>◯ Disagree<br>◯ Totally disagree

3.1.5	***If 'Have you read the conversation aid?' is equal to 'Yes' answer this question:***<br>The conversation aid is not neutral.	◯ Totally agree<br>◯ Agree<br>◯ Do not agree, do not disagree<br>◯ Disagree<br>◯ Totally disagree
3.1.6	***If 'Have you read the conversation aid?' is equal to 'Yes' answer this question:***<br>The conversation aid made me insecure.	◯ Totally agree<br>◯ Agree<br>◯ Do not agree, do not disagree<br>◯ Disagree<br>◯ Totally disagree
3.1.7	***If 'Have you read the conversation aid?' is equal to 'Yes' answer this question:***<br>The information in the conversation aid made me scared or sad.	◯ Totally agree<br>◯ Agree<br>◯ Do not agree, do not disagree<br>◯ Disagree<br>◯ Totally disagree
3.1.8	***If 'Have you read the conversation aid?' is equal to 'Yes' answer this question:***<br>The conversation aid is understandable.	◯ Totally agree<br>◯ Agree<br>◯ Do not agree, do not disagree<br>◯ Disagree<br>◯ Totally disagree
3.1.9	***If 'Have you read the conversation aid?' is equal to 'Yes' answer this question:***<br>The conversation aid helped me to form an opinion about care decisions.	◯ Totally agree<br>◯ Agree<br>◯ Do not agree, do not disagree<br>◯ Disagree<br>◯ Totally disagree
3.1.10	***If 'Have you read the conversation aid?' is equal to 'Yes' answer this question:***<br>The conversation aid helped me feel better prepared to talk to my physician about care decisions.	◯ Totally agree<br>◯ Agree<br>◯ Do not agree, do not disagree<br>◯ Disagree<br>◯ Totally disagree
3.1.11	***If 'Have you read the conversation aid?' is equal to 'Yes' answer this question:***<br>The conversation aid helped me feel better prepared to talk to my family or friends about care decisions.	◯ Totally agree<br>◯ Agree<br>◯ Do not agree, do not disagree<br>◯ Disagree<br>◯ Totally disagree

3.1.12	***If 'Have you read the conversation aid?' is equal to 'Yes' answer this question:***<br>The conversation aid does not take too much time.	○ Totally agree<br>○ Agree<br>○ Do not agree, do not disagree<br>○ Disagree<br>○ Totally disagree
3.1.13	***If 'Have you read the conversation aid?' is equal to 'Yes' answer this question:***<br>I give the conversation aid a...	○ 1 ○ 2 ○ 3 ○ 4 ○ 5 ○ 6 ○ 7<br>○ 8 ○ 9 ○ 10
3.1.14	***If 'Have you read the conversation aid?' is equal to 'Yes' answer this question:***<br>Do you have other comments, tips or suggestions about the conversation aid?	

Survey 'Patient's questionnaire (intervention)'

Patient's questionnaire (intervention) - Health perception and mood.

Number	Question	Answers
	The following questions are about how you assess your health. The answers help to keep track of how you are feeling and how well you are able to perform your usual activities.	
4.1	How do you view your health?	◯ Good<br>◯ Reasonable<br>◯ Neutral<br>◯ Moderate<br>◯ Bad
4.2	How do you experience your health?	◯ 1 ◯ 2 ◯ 3 ◯ 4 ◯ 5 ◯ 6 ◯ 7<br>◯ 8 ◯ 9 ◯ 10
4.3	How do you experience your quality of life?	◯ 1 ◯ 2 ◯ 3 ◯ 4 ◯ 5 ◯ 6 ◯ 7<br>◯ 8 ◯ 9 ◯ 10
4.4	How much are you limited in your work or other daily activities by your physical health?	◯ 1 ◯ 2 ◯ 3 ◯ 4 ◯ 5 ◯ 6 ◯ 7<br>◯ 8 ◯ 9 ◯ 10
4.5	How much are you limited in your work or other daily activities by your emotional health?	◯ 1 ◯ 2 ◯ 3 ◯ 4 ◯ 5 ◯ 6 ◯ 7<br>◯ 8 ◯ 9 ◯ 10
4.6	In the past 2 weeks, how often have you suffered from one or more of the following problems?<br>Little interest or pleasure in activities.	◯ Not at all<br>◯ Multiple days<br>◯ More than half of the days<br>◯ Almost every day
4.7	In the past 2 weeks, how often have you suffered from one or more of the following problems? Feeling down, depressed, or desperate.	◯ Not at all<br>◯ Multiple days<br>◯ More than half of the days<br>◯ Almost every day
4.8	Can you turn to your neighbors if you need help?	◯ Not at all<br>◯ Barely<br>◯ Maybe<br>◯ Reasonable<br>◯ Certainly

4.9	How many people can you contact if you have serious problems??	◯ None<br>◯ 1-2 people<br>◯ 3-5 people<br>◯ 6 or more people
4.10	How much involvement and interest do people show in what you do?	◯ A lot<br>◯ A little<br>◯ Unclear<br>◯ Few<br>◯ No

Patient's questionnaire (intervention) - General questions

Number	Question	Answers
5.1	What is your marital status?	☐ Single<br>☐ Living together<br>☐ Married<br>☐ Divorced<br>☐ Widow or widower
5.2	Do you have any children?	◯No<br>◯Yes
5.2.1	If 'Do you have any children?' is equal to 'Yes' answer this question:<br>How many children do you have?	[ ] children
5.3	What is your highest level of completed education?	☐ Primary education (Basisonderwijs)<br>☐ Secondary education (Vmbo of onderbouw havo-vwo)<br>☐ Middle education (MBO of bovenbouw havo-vwo)<br>☐ Higher education (HBO of WO bachelor)<br>☐ Higher education (Dutch: WO master)
5.4	Are you currently working?	◯ No<br>◯ Yes
5.4.1	***If 'Are you currently working?' is equal to 'Yes' answer this question:***<br>How many hours per week do you work on average?	[ ]
5.4.2	***If 'Are you currently working?' is equal to 'No' answer this question:***<br>Explanation:	◯ Retired<br>◯ Disabled<br>◯ Unemployed<br>◯ Other

This is the end of the questionnaire. This is also the last part of this research. If you have any questions about the study as a result of the questionnaire, you can contact the researchers via (e-mail address removed), or the independent physician, via (e-mailaddress removed) or via (telephone number removed).
