# Supplementary material for: The effect of physician training and patient education on the discussion of care decisions at the internal medicine outpatient clinic
Source: BMC Health Serv Res. 2022 Dec 22;22:1569. doi: 10.1186/s12913-022-08901-7 (PMC9773541; doi:10.1186/s12913-022-08901-7)
Supplement: Supplementary file 2 — Additional file 2. [file 12913_2022_8901_MOESM2_ESM.docx]

Supplementary Appendix 1. Additional information about the physicians’ training.

*E-learning module*The e-module is in Dutch, and consist of videos of different professionals (e.g. medical ethic, several physicians, legal advisor) and patients to illustrate the importance of the discussion of care decisions. Besides, background information is provided (e.g. outcomes after resuscitation, a webinar about cultural sensitive communication), common pitfalls are discussed (reasons given for not discussing care decisions and why there are no excuses not to do so) and some physicians give ‘golden tips’, why and how they discuss care decisions. Finally, six videos of example cases in various settings (e.g. internal medicine outpatient clinic, general practitioner, pre-operative screening) in which a physician discusses care decisions with a simulated patient (i.e. an individual trained to act as a real patient) can be viewed.

*Pilot test*Prior to this study, a pilot test of the e-learning module was performed among residents. 18 residents (33% male, education year 1-6, median 3 years) participated in this pilot. 83% of the residents thought the e-module was (very) clear. 89% indicated that after the training they had sufficient insight into the usefulness and necessity of discussing treatment wishes and limitations and 55% indicated that they had sufficient tools to do so afterwards.

*Training with simulated patients*Physicians were trained in couples (one resident and one medical specialist) combined with one simulated patient. The training consisted of four cases, two in an internal medicine outpatient clinic setting and two in an emergency department setting. Each doctor practiced the ‘doctor-role’ in two cases, and observed and provided feedback in the other two cases. Additionally, the simulated patient provided feedback. Both practicing and observing and providing feedback were intended to improve communication skills in discussing care decisions.
