# Supplementary material for: The effect of physician training and patient education on the discussion of care decisions at the internal medicine outpatient clinic
Source: BMC Health Serv Res. 2022 Dec 22;22:1569. doi: 10.1186/s12913-022-08901-7 (PMC9773541; doi:10.1186/s12913-022-08901-7)
Supplement: Supplementary file 3 — Additional file 3. [file 12913_2022_8901_MOESM3_ESM.docx]

Supplementary appendix 3. Multilevel mixed model

***Mean patient satisfaction***

| **Variable** | **Effect size** | **95% Confidence Interval** | **P-value** |
| --- | --- | --- | --- |
| Intercept | 8.059 | 6.687 to 9.430 | <0.001 |
| *Fixed effects* |  |  |  |
| Patient’s age | -0.102 | -0.030 to 0.009 | 0.305 |
| Patient’s gender (male versus female) | -0.333 | -0.750 to 0.083 | 0.116 |
| Charlson Comorbidity Index | 0.091 | -0.007 to 0.189 | 0.068 |
| Quality of life | 0.181 | 0.057 to 0.305 | 0.005 |
| Physician’s gender (male versus female) | -0.424 | -0.868 to 0.020 | 0.060 |
| Level (resident versus specialist) | -0.275 | 0.730 to 0.179 | 0.216 |
| Physician training (trained versus untrained) | -0.043 | -0.483 to 0.398 | 0.840 |
| Patient conversation aid (intervention versus control) | -0.142 | -0.558 to 0.273 | 0.499 |
| *Random effects* | **Estimate** | **Standard error** |  |
| Physician training | 0.008 | 0.075 |  |
| Residual variance | 1.137 | 0.165 |  |

***Patient-doctor relationship***

| **Variable** | **Effect size** | **95% Confidence Interval** | **P-value** |
| --- | --- | --- | --- |
| Intercept | 34.8877 | 26.963 to 42.790 | <0.001 |
| *Fixed effects* |  |  |  |
| Patient’s age | -0.038 | -0.151 to 0.076 | 0.514 |
| Patient’s gender (male versus female) | -1.464 | -3.867 to 0.940 | 0.230 |
| Charlson Comorbidity Index | -0.118 | -0.681 to 0.445 | 0.678 |
| Quality of life | 1.063 | 0.346 to 1.780 | 0.004 |
| Physician’s gender (male versus female) | -0.929 | -3.317 to 1.458 | 0.442 |
| Level (resident versus specialist) | -2.062 | -4.449 to 0.325 | 0.090 |
| Physician training (trained versus untrained) | 1.279 | -1.044 to 3.602 | 0.278 |
| Patient conversation aid (intervention versus control) | -0.450 | -2.850 to 1.950 | 0.711 |
| *Random effects** | **Estimate** | **Standard error** |  |
| Residual variance | 38.221 | 4.997 |  |

* physician training was redundant as a random effect in this model, and therefore removed.

***Shared-decision-making***

| **Variable** | **Effect size** | **95% Confidence Interval** | **P-value** |
| --- | --- | --- | --- |
| Intercept | 58.985 | 39.092 to 78.877 | <0.001 |
| *Fixed effects* |  |  |  |
| Patient’s age | 0.131 | -0.151 to 0.413 | 0.358 |
| Patient’s gender (male versus female) | -7.724 | -13.755 to -1.693 | 0.013 |
| Charlson Comorbidity Index | 0.746 | -0.677 to 2.168 | 0.301 |
| Quality of life | -1.202 | -2.951 to 0.547 | 0.179 |
| Physician’s gender (male versus female) | 8.952 | 1.364 to 16.540 | 0.023 |
| Level (resident versus specialist) | 4.291 | -4.643 to 13.225 | 0.299 |
| Physician training (trained versus untrained) | -0.235 | -8.894 to 8.424 | 0.951 |
| Patient conversation aid (intervention versus control) | -0.009 | -5.956 to 5.937 | 0.998 |
| *Random effects* | **Estimate** | **Standard error** |  |
| Physician training | 28.359 | 36.268 |  |
| Residual variance | 216.889 | 35.346 |  |
